# Situation awareness in intensive care unit nurses: A qualitative directed content analysis

**DOI:** 10.3389/fpubh.2022.999745

**Published:** 2022-10-14

**Authors:** Chiman Ghaderi, Roghayeh Esmaeili, Abbas Ebadi

**Affiliations:** ^1^Student Research Committee, School of Nursing and Midwifery, Shahid Beheshti University of Medical Sciences, Tehran, Iran; ^2^Department of Medical-Surgical, School of Nursing and Midwifery, Shahid Beheshti University of Medical Sciences, Tehran, Iran; ^3^Behavioral Sciences Research Center, Life Style Institute, Baqiyatallah University of Medical Sciences, Tehran, Iran; ^4^Nursing Faculty, Baqiyatallah University of Medical Sciences, Tehran, Iran

**Keywords:** Situation awareness, intensive care unit nurses, intensive care unit, directed content analysis, Endsley's model

## Abstract

**Background:**

Situation awareness (SA) is an essential cognitive construct to create positive patient safety outcomes. SA of the nurses in the intensive care unit (ICU), where conditions may change rapidly, is particularly important. The present study aimed to explain the perception and experience of SA in ICU nurses based on Endsley's SA model.

**Materials and methods:**

This qualitative directed content analysis was conducted on nurses in six hospitals in Tehran, Iran, from December 2020 to July 2021. Twenty-seven ICU nurses were selected using purposive sampling. Data were collected by semi-structured interviews and field observations. The data were analyzed based on the Elo and Kyngas method modified by Assarroudi et al. COREQ checklist was used to report the research.

**Results:**

The concept of SA in ICU nurses, based on Endsley's model, includes perception of patients' clinical cues, perception of the human environment, perception of the physical environment, and perception of the organizational environment as generic categories of the perception of the elements in the environment. SA in ICU nurses also includes the main categories of comprehension the current situation through a sense of salience and interpretation of cues and projection the future situation through the prediction of patient status into the near future and environmental foresight.

**Conclusion:**

Findings have further developed the concept of SA in ICU nurses based on Endsley's SA model. The insights and knowledge gained from this study can be useful for future practice, education, and research on SA among ICU nurses.

## Introduction

Endsley (1), an aviation and military theorist, defined Situation awareness (SA) as “the perception of the elements in the environment in a volume of time and space, the comprehension of their meaning, and the projection of their status into the near future” (1). In the mid-1990s, the value of this concept was first introduced into anesthesiology with a direct emphasis on patient safety in health care, and its application then spread to other health professions, including nursing (2–4).

SA is an essential cognitive construct to create positive outcomes for patient safety (5). SA is the foundation of the decision-making process in the clinical setting and is a key feature in enhancing accountability and clinical management and reducing medical errors (6, 7). This is especially the case in ICUs due to the wide variety of hospitalized patients, the use of special treatment methods, the need for immediate and high-risk decisions, and the use of specific medical and pharmaceutical tools (8–10). The ability to interpret key information and make predictions for a dynamic situation is a hallmark of the higher levels of SA.

Emerging evidence indicates that inadequate SA may be responsible for half of the adverse events in hospital settings due to constant failure to recognize and respond to signs of patient deterioration in a timely manner (11, 12). As the largest group of care providers who perform important tasks such as patient assessment and 24-h care, nurses work at the forefront of the health care system, where they can identify vulnerabilities in the system and maintain patient safety (13, 14). The presence of SA in the nurse is a prerequisite that should be clearly identified, defined, and operationalized for safe care (2, 6, 14). Compared to other disciplines, this concept is relatively new in nursing and is not yet well explored (6). In their study, Walshe et al. (15) emphasized that more studies connected with the concept of SA have to be conducted in nursing (15).

### Purpose

Lack of clarity in this key concept can lead to its devaluation and incorrect use in the patient care process (2). There is a clear need to understand nurses' perspectives and experiences using a qualitative approach based on a naturalistic paradigm to further identify and clarify the concept of nurses' SA in the ICU. Therefore, this study aimed to explain the perception and experience of SA in ICU nurses.

### Theoretical framework

In Endsley's theory, SA has three levels: perception, comprehension, and projection (1, 16). Level 1 is the perception of the elements in the environment and will be the basis for levels two and three. Cues are perceived by one or more of the five senses. At level 2, the person begins to distinguish patterns, recognize the connection between cues and goals, and create a unified picture of the situation. In level 3, based on the understanding achieved in level 2, the person begins to predict; this level is the highest level of SA (1, 16). We chose this theory because it provides a universal model and the most widely accepted definition of situational awareness. Endsley's model is the most commonly used in health care. More importantly, it emphasizes the possibility of abstraction at all three levels, which is consistent with the objectives of this study (15).

## Method

This study was conducted using the directed content analysis method. Directed content analysis is used to validate and develop the theories related to a studied phenomenon (17). The Consolidated criteria for reporting qualitative research (COREQ) checklist was utilized in this study to provide an exhaustive report (Supplementary material 1) (18).

### Participants

Twenty-seven nurses working in ICUs of six teaching hospitals in Tehran, Iran, with maximum diversity in terms of age, gender, work experience, and level of education, were included in the study. The participants were selected based on purposive sampling, a suitable method for qualitative research (19). Nurses employed in different work shifts with at least a bachelor's degree were included in the study. Nursing managers, supervisors, and ward managers who were not directly involved in patient care were not included. The ICU sizes in this study ranged from 5 to 26 beds.

According to Sandelowski's (20) suggestion, the sample size should be more qualitative than quantitative, and its size should be determined by data saturation (20). We continued data collection until saturation was achieved. In general, saturation refers to a situation in which further information is not obtained by continuing to collect data (21). In this study, data saturation was obtained after 22 interviews, but another five interviews were conducted to ensure no new information is added to the study.

### Data collection

Data collection was done from December 2020 to July 2021 through one-on-one in-depth semi-structured interviews with open-ended questions. Interviews were conducted with a conversational and emphatic approach (19). All interviews were conducted by the project PhD student who had received complete training on conducting qualitative studies and interviews using the interview guide (Table 1). During the interviews, exploratory questions, such as “What do you mean?” or “Please explain more,” were used to increase the interviewer's understanding of the participants' experiences.

**Table 1:** Interview guide.

Perception	When you hand over your patient at the beginning of the shift, what assessments and evaluations do you do? How about in the ward? What do you pay attention to? What do you monitor in your patient during the shift?
Comprehension	What is the purpose of this assessment and monitoring? How do you diagnose a patient's condition based on their cues and symptoms?
Projection	Can the patient's problems be predicted in the ICU? Can you explain how? When you take over the shift, have you ever thought, “what a shift it is going to be today!” Can you talk a little bit about this?
Closing questions	In your opinion, what does it mean when they say an ICU nurse should know what is going on around them during care provision? Before we end the interview, is there anything I missed that you would like to add?

At the end of each interview, the participant was asked, “In your opinion, what does it mean when they say an ICU nurse should know what is going on around them during care provision?” to allow them to think about their perceptions, their efforts to understand their situation and their role as an ICU nurse.

After the end of each interview, the participant's personal information, including age, gender, work experience, place of work, level of education, and position in the ward, were entered into an information form. Interviews ranged from 18 to 87 min (971 min in total).

This study used unstructured observation alongside the interviews to collect data. The researcher referred to the ICUs in 15 day and night shifts (at the beginning, during, and end of shifts), each for 2–3 h (40 h in total), and observed the daily activities of the nurses, their care provision, interactions of nurses with each other, their patients, and their colleagues. Close observation enables the researcher to closely follow the everyday experiences of the nurses and validate them (19). The environmental conditions of the ward and field observations were recorded in all visits and included in the data analysis.

### Data analysis

The transcripts of the interviews were analyzed according to the eight steps presented by Assarroudi et al. (22) for organizing the data (22). Data analysis began at the same time as data collection. The analysis unit in this study was the transcript of the interviews and the interviewer's observations in the field.

The main steps of the directed qualitative content analysis included reading the text of the interviews several times to immerse yourself in the data, entering the text of the interviews into MAXQDA software version 10 to manage the data, developing a formative categorization matrix to place the codes into predetermined categories based on the three levels of Endsley's (1) model, extracting semantic units as sentences or paragraphs from interview texts and specifying the initial codes, placing the codes based on common characteristics of subcategories, placing the subcategories into generic categories of the coding matrix, creating new generic categories for those subcategories that did not fit into any of the generic categories, constantly examining the relationship between the new generic categories and the main categories, selecting participants' contributory quotes for each extracted feature, and providing an operational definition for SA based on the combination of participants' perceptions and the extracted latent content.

### Ethical considerations

This qualitative study is a part of the PhD dissertation in nursing, approved by the Ethics Committee of Shahid Beheshti University of Medical Sciences with the code IR.SBMU.PHARMACY.REC.1398.032. Informed consent to participate in the study was obtained from individuals. The participants determined the time and place of the interview and were asked for permission for their voices to be recorded.

### Trustworthiness

Speziale et al. (19) criteria, including credibility, dependability, transferability, and confirmability to ensure trustworthiness, were considered. The research team reviewed the interviews after being coded to achieve peer review. External review was done by two nursing assistants outside the research team, and participant reviews were also done randomly by three participants to confirm the accuracy of the results. Purposive sampling with maximum diversity helps data transferability. In addition to the complete report of all the steps performed and the clear description of the analysis process, participants' quotations were also recorded to indicate that the findings are relevant to the data.

## Results

Twenty-seven nurses working in ICUs participated in this study. Participants included 11 men and 16 women aged 26–50 years and work experience of 7 months to 26 years from different ICUs (Table 2).

**Table 2:** Characteristics of participants.

**Participants**	**Age (year)**	**Gender**	**Education**	**Professional title**	**Type of ICU**	**Working experience in ICU (year)**
N1	34	Female	PhD	Supervisor	General	4
N2	42	Female	Bachelor	Supervisor	Neurosurgery	3
N3	48	Female	Master	Supervisor	Respiratory	6
N4	28	Female	Bachelor	RN	General	5
N5	45	Female	Bachelor	RN	Respiratory	8
N6	29	Male	Bachelor	RN	General	1
N7	43	Female	Master	Supervisor	General	10
N8	37	Female	Master	RN	General	6
N9	26	Female	Bachelor	RN	General	2
N10	33	Female	PhD	RN	Respiratory	5
N11	50	Male	Bachelor	RN	Toxicology	20
N12	35	Male	Bachelor	Supervisor	Neuro surgical	10
N13	49	Female	Bachelor	RN	Toxicology	26
N14	33	Male	Bachelor	RN	Cardiac surgery	6
N15	33	Female	Master	RN	General	7 (Month)
N16	26	Female	Bachelor	RN	General	3
N17	43	Male	Bachelor	RN	Toxicology	10
N18	38	Male	PhD	RN	Cardiac surgery	7
N19	38	Male	Bachelor	Supervisor	Cardiac surgery	7
N20	31	Male	Master	Supervisor	General	5
N21	28	Male	Bachelor	RN	Toxicology	2
N22	47	Female	Bachelor	RN	Toxicology	8
N23	45	Male	Bachelor	RN	Toxicology	23
N24	37	Female	Bachelor	RN	Covid-19	10
N25	35	Female	Bachelor	RN	Cardiac surgery	10
N26	37	Female	PhD	RN	General	5
N27	32	Male	Master	RN	General	3

In the analysis of interviews, 1,128 primary codes were extracted. After merging similar codes, 71 primary codes with a frequency of 780 were obtained. After their integration, finally, 19 subcategories were obtained, which were placed in 8 generic categories and the three main categories of “the perception of the elements in the environment,” “comprehension of the current situation,” and “projection of future status,” according to Endsley's theory of situational awareness, which will be explained separately. Table 3 shows the main categories, generic categories, and subclasses.

**Table 3:** Main categories, generic categories, and subcategories of SA in ICU nurses.

**Main categories**	**Generic categories**	**Subcategories**
The perception of the elements in the environment	The perception of the patient's clinical cues	Initial patient assessment Continuous patient assessment Comprehensive attention to the dimensions of patient health Attention to patient handover
	The perception of the human environment	Simultaneous attention to patients Dynamic interaction with the team
	The perception of the physical environment	Awareness of patient and ward equipment Attention to the environmental conditions of the ward.
	The perception of the organizational environment	Awareness of existing care protocols and measures Awareness of the duties and roles of the nurse
Comprehension of the current situation	Sense of salience	Ensuring the accuracy of the cues Recognizing the cues
	Analysis and interpretation of cues	Critical review of symptoms and cues Problem diagnosis
Projection the future situation	Prediction of patients' status in the near future	Projection of the patient's situation Projection for action Projection of possible complications
	Environmental foresight	Expecting a sudden change of circumstances contrary to the expected conditions. Predicting the shift conditions when entering the ward

### Main category 1: The perception of the elements in the environment: Building blocks of nurse's situational awareness

The most basic and essential activity to know what is going on around us is to get information about that situation. The patient is the main component of the clinical situation. The ICU nurses pointed out that by correctly identifying the patient, reviewing the patient's document, communicating with the patient's relatives, and accurately assessing the patient, they perform the initial assessment of the patient at the beginning of the shift and collect fixed and basic patient information. You only need to collect fixed information once to be retrieved later whenever necessary.

“*The patients stay here for a long time, more than just a few days; once you read the file, you will know everything. What was their complaint? What is their history? It is very important to have a first impression of the patient.”* N5

Information such as vital signs and cues of changes in a patient's condition is dynamic information. For dynamic information, you need to be constantly monitoring the patient. Nurses stated that they were continuously evaluating the patient by being present at the patient's bedside, using their senses and monitoring devices, and communicating with the patient or other members of the treatment team.

“*The best way is for the nurse to constantly be beside the patient and do everything there because this way they will definitely notice the smallest issue.”* N4

“*Patient's vital signs should be monitored constantly. Although every patient here is being monitored by the systems, we should be attentive that the pulse oximetry is connected and the chest leads are correctly placed.” N5*

“*The patients themselves tell us if they are feeling better or not; but the thing is, not every patient in the ICU is conscious although we sometimes do have conscious patients.” N7*

In addition to these evaluations, ICU nurses emphasized comprehensive attention to health dimensions by having a holistic view, examining physiological needs, communicating with the patient, and paying attention to the psycho-social dimension of patients in the ICU and during patient handover.

“*When I notice that the patient is anxious, I'll try to talk to them and see if I can make them feel better by talking. Anyway, some people are very dependent on their families, so the patient's companion can come to their side and calm them for a few minutes. If I see that this works, I'll definitely do it.” N12*

Patient handover involves the transfer of responsibility for patient care to another person, shift, ward, or hospital. Accurate information transfer is the main goal of the patient handover process.

“*In our ward, the ISBAR technique is used for patient handover. The previous shift provides us with the patient's condition, background, the assessment of the previous shift nurse, and the recommended course of action. Accurate patient handover is of particular importance.”* N20

When talking about the patient's environment, the human, physical, and organizational aspects must be considered. The human environment includes all health care providers and other patients. Nurses said that during the shift, in addition to paying attention to their patients, they should be somewhat aware of the condition of other patients in the ward.

“*When the head-nurse hands over the shift, I usually wonder how complicated this patient's condition is now; for example, how much can they challenge us during the shift?”* N18

Nurses noted that in addition to paying attention to patients, they try to have dynamic communication with the team through sharing patient information with the team, communicating and cooperating with each other, and paying attention to things other treatment team members do.

“*The relationship among treatment staff is so important. For example, the situation should not be such that the nurse and the physiotherapist do their jobs separately. For example, if the physiotherapist is asked to perform chest physiotherapy and the patient has high ICP, they should be told to restrict some movements*.” N7

The physical environment around the patient includes devices and equipment and other environmental conditions such as light, sound, and ambient temperature. Nurses said that due to many pieces of equipment and devices for evaluating and caring for the patient in the ICU, a nurse should be aware of the equipment, its storage, and its usage. At the beginning of each shift, they have to check patients' bedside equipment and connections.

“*Considering that the patients are intubated, it is very important that the tube is fixed in its position and the ventilator's settings are correct; if there is an IV line then it should be checked, and also, the CV line and NG tube should be examined. I'll definitely check these.” N22*

Some nurses said that nurses should also pay attention to light, sound, ventilation, and cleaning.

“*If the patient is resting, I turn down the light or warn others about the noise so it is quieter.”* N10

The organizational environment includes protocols, care measures, and the duties and roles of the nurse. Nurses said that there are specific medical protocols and instructions in the ICU to follow in specific scenarios.

“*In the ICU, all treatment protocols for common poisonings are put up on the wall in the ward in the form of large posters. In other ICUs, posters for the cardiopulmonary resuscitation algorithm, how to assess the consciousness level with different criteria, blood transfusion instructions, etc. are put up on the wall.”* Field note Hospital 1

The nurse and every other member of the team have specific duties and responsibilities for the care and treatment of the patient. Participants stated that the nurse should be aware of their role in the treatment team because due to their constant presence in the ward and patient bedside, they are aware of all the patient's issues and can notice changes and the patient's cues sooner than other treatment team members.

“*I daresay that because we are a neurosurgery center, the first people that notice the GCS drop of the patient are the nursing staff.” N2*

They also said that the nurses are responsible for providing safe and high-quality care to ICU patients.

“*We do all this so that we don't miss anything about the patient, and there is no necessary measure that we fail to take. The patient's safety should be at the highest level, and they should receive high-quality and safe care.”* N19

### Main category 2: Comprehension of the current situation: A sense of salience and analysis of cues to achieve a diagnosis

Once you have gathered the information, you need to go beyond it. The next step is to be aware of the situation and comprehend, analyze, or interpret the gathered information. At this stage, a nurse thinks about the information, reasons, and evaluations, and by critically examining the cues, they finally reach a judgment and diagnosis.

Participants said that the nurse verifies the cues after checking the correctness of the observed cues and comparing the observed cues with the patient's basic information.

“*For example, my patient has a drop in O2 Sat. I will check their pulse oximeter and see if it is working; I check to see if their limbs are cold, and I connect the oximeter to his ear to see if the O2 Sat. is right.”* N8

In order to notice the cues, a nurse should know the specific warning signs so that they can distinguish what is normal and what is abnormal. They should know the cues of common complications in the ICU and report them in a timely manner.

“*Here, the patients' pupils are very important because, through the changes in consciousness and pupil [size], we can notice bleedings that can occur after surgery.”* N1

Nurses said that they examine the possible causes of the changes in the patient's condition, prioritizing the patient's main problem, and re-evaluating all systems.

“*Most of the time, we encounter symptoms that can be similar in different problems. For example, when the capnograph of one of my patients shows that CO2 levels are increasing, the first impulse for me is to say that it's because of COPD, because all our patients are admitted with respiratory issues. However, this rise can be due to fever or hyperthyroidism or …”* N5

After critically examining the cues, a nurse puts all the symptoms together to identify the relationship between them by using their knowledge and experience or by reporting the patient's symptoms so that the health care team may diagnose the problem.

“*... in the tamponade cases that I mentioned, the patient's rhythm goes up, the pressure goes down, and the way they breathe changes. They may even have a state of lethargy in terms of consciousness. Many symptoms help us to recognize and identify the problem”* N25

### Main category 3: Projection of future situation: Prediction of patients' status in the near future and environmental foresight

The last cognitive activity that complements situation assessment and awareness is thinking about the future. This ability refers to the use of the obtained conceptual information to extrapolate the situation in the near future. Nurses emphasized that they should make the necessary predictions about the patient's possible condition and the effects of the patient's history (underlying diseases, previous medications, and previous habits) on the patient's condition, in order to take the measures necessary for the patient's near future and keep nursing care ahead of the patient's condition.

“*A good nurse should always be one step ahead of clinical symptoms. This means that for example, they should be able to predict shock before it happens. The reason for this, is that when the patient is in shock, it will be much harder to bring him out of shock. You can definitely do much better before something happens, and this means that there will be less damage.” N7*

“*Patients who are drug users have very bad prognoses, and they are very restless when they are admitted to the ICU. These patients fight hard when they are connected to the ventilator and have withdrawal signs; they may even fall out of the bed”* N4

“*At about 10:30 a.m., a 72-year-old patient in bed 2 with a diagnosis of CVA is not hemodynamically stable. A nurse tries to adjust the syringe pump to deliver medication, and a resuscitation trolley has been brought to the patient's bedside.”* Hospital 2 field notes

They also stated that the patient's admission to the ICU and the pharmacological and medical treatments always have complications that a nurse should anticipate.

“*... It is very common for patients to develop emphysema one or two days after surgery before or after the drain is removed, so we check them for emphysema.”* N14

In visualizing the future situation and the projection of the patient's near future, the ICU nurse should also have environmental foresight based on the information gathered and prior knowledge of the work environment. Sometimes, due to the complexity of patients' conditions in the ICU, a nurse must expect the patient's condition to change contrary to expectation, sometimes instantaneously.

“*I cannot say that everything can be predicted because I also believe in miracles. Sometimes you see a patient has a very bad condition, and everyone says he will die, but you see that they become stable.”* N10

“*The ICU is a sensitive department and the patients should be monitored every second because much of these incidents happen in an instant and the nurse should be alert at all times.”* N12

The nurses said that a nurse can sometimes predict the conditions of the shift at the beginning of the shift and when entering the ward, relying on intuition, the stability or instability of patients in the ward, the appearance of the ward, relationships between team members and colleagues, and the new patient admissions in the ward.

“*When we enter the ward, we see that the ward is a mess, one nurse is preparing a patient for a CT scan, another nurse is trying to insert a CV line,... we realize that we are not going to have a good shift.”* N16

“*I really don't know, It's a feeling. I can't tell you exactly what it is. But it's a feeling that when I enter the department I can tell that it's going to be a quiet night and eventually, it will be.”* N20

## Discussion

Dimensions of SA among intensive care nurses based on Endsley's theory include the perception of the elements in the environment, comprehension of the current situation, and projection of the situation into the future; the findings of the study help clarify these abstract dimensions by presenting the characteristics of these dimensions in ICU nurses.

After analyzing the concept of SA in nurses using the Walker and Avant method (2011), Fore and Sculli (2) provided a definition very similar to Endsley's (2). Conducting the present study qualitatively and relying on the theoretical framework helped extract more context-based information about this concept.

In this study, perception was recognized as an essential part of situational awareness, and most of the subcategories and extracted codes were related to this level of SA. The ICU nurses in this study understood the patient's clinical signs through initial and continuous patient evaluation, comprehensive attention to the dimensions of patient health, and attention to patient handover. A nurse simultaneously pays attention to patients, establishes a dynamic interaction with the team, and obtains information on the equipment of the patient and the ward. The nurse also monitors the environmental conditions and physical environment of the ward and keeps in mind the existing protocols and care measures, their duties and roles, and the organizational environment, as other important elements in the clinical environment.

Sitterding et al. (23) in their study aimed at analyzing the concept of SA in nursing, defined SA as a dynamic process in which a nurse in any clinical environment receives information about the patient and their environment, then understands their meanings depending on the patient, and predicts the necessary interventions (23). Similar to the present study, Sitterding et al. (23) made the collection of patient information the main focus at the perception level.

However, in their study, they referred to the general concept of environment. In contrast, in the present study, in addition to the patient, the human environment, physical environment, and organizational environment were also obtained as other environmental elements in the ICU. Further studies are necessary to examine how different the elements obtained in other wards and situations are from those obtained in this study.

In an ICU, the environment consists of a variety of equipment and a lot of staff. The physical environment, as part of nursing is already embedded in the earliest nursing theories. The physical environment is important for nurses to maintain accessibility to and visualization of the patient (24). Petersson et al. (25) reported that the physical environment, including the medical equipment, workstations, and beds, highly affects ICU nurses' care (25). Also; Gharaveis et al. (26) reported that layout design, visibility, and accessibility levels are the most cited aspects of design that can affect teamwork and the level of communication in healthcare settings (26). Similar to the results of these studies, the nurses of our study also referred to the physical environment, including medical equipment, the location of the patient's bed, lighting, and ventilation for a better understanding of the situation.

Kvande et al. (27) reported in a phenomenological study that nurses in the ICU acquire patients' cues using their senses, measurements, and intuition (27). In the present study, the nurses did not mention intuitive awareness of the patient's symptoms, but in the projection part, they mentioned that they intuitively felt how the shift would go at the beginning of the shift.

The other studies also reported the presence of intuition in care providers in understanding the symptoms and projecting the situation (28, 29). In our study, nurses noted that when they feel the shift will be complicated with their intuition, they try to be more careful in planning and setting care goals from the very beginning.

All health care providers working in acute environments must be able to respond quickly to patient deterioration. Endsley referred to the challenge of obtaining and using relevant data from a data-rich environment as an information gap (30). The present study's findings can be a model to show what information nurses pay attention to in similar environments and how and from what sources they acquire that information.

Research in aviation, driving, and health care has shown that most errors occur at level 1 of SA (10, 31, 32). Endsley emphasizes the importance of design, for example, improving monitor connectors and cockpit design to increase perception in aviation (33). However, the importance of designing the physical space of the ward and using the appropriate equipment can also be extended to the ICU.

Although much of the data in health care comes from other people, patients, caregivers, and colleagues, it cannot be improved through design, but targeted educational interventions may improve SA. We will be able to provide techniques to strengthen this level of SA through increasing awareness of what SA is and how it affects performance, and teaching strategies to improve data collection, focus, situational sensitivity, communication, and coordination.

In their review, Massey et al. (34) point out that recognizing and responding to patient deterioration is complex, challenging, and multifaceted and requires the identification and combination of multiple symptoms of patients. Knowing the patient led to a sense of salience and an ability to recognize aspects of the patient's clinical situation, which is important for ward nurses' judgment (34).

In this study, nurses said that prior knowledge of the patient, comparison of current cues with baseline cues, and recognition of warning signs of the disease lead to a sense of salience and importance of the cues, and, finally, diagnosis of the patient's current situation.

The sense of salience is an ability to recognize the subtle changes in a situation acquired through understanding and knowing the patient's deep background (35). In a thematic study, Lavoie et al. (36) have identified surveillance, recognition, referral, and response as four processes related to patient deterioration in the ICU that require careful evaluation and surveillance during the monitoring phase. In the recognition phase, the data are interpreted, and in the referral phase, nurses communicate with other clinicians by conveying the data to determine the appropriate therapeutic interventions, which is confirmed by the results of the present study (36). Because the nurses could not perform some interventions and prescribe medications themselves, they said that a physician should make a final diagnosis.

In addition to problem diagnosis, there is a concept of nursing diagnosis in the nursing literature. Nurses use nursing diagnoses to accurately share patient health problems, high-risk situations, and patient readiness for health promotion (37). However, in the present study, nurses did not mention this concept, which is probably due to the lack of awareness of the nurses who participated in the present study of the importance of this concept in care. In field observations, it was also observed that nursing diagnoses were mentioned routinely and in many similar cases in nursing reports.

The study results by Juvé-Udina et al. (38) indicate that the high number of nursing diagnoses in the ICU might reflect poor prioritization and a linear decision-making process, and problem perception regardless of the complete situation of the patient (38).

Future studies need to investigate the relationship between nursing diagnoses for the patient and SA in ICU nurses.

Level 3 is the highest level of SA and is the level at which health care providers project the patient's expected condition, which is critical to achieving treatment goals through managing the treatment process and adequacy of resources. ICU nurses work in a very complex and technical environment, and some conditions are highly unpredictable and require the ability to accurately identify and change priorities rapidly (27).

However, there are criteria for predicting the patient's deterioration and mortality rate. In the present study, ICUs use the Failure Organ Sequential Assessment (SFOA) and APACHE II criteria to predict the mortality and severity of patients' conditions. However, in the worksheets, these cases were evaluated by an ICU physician, and participants did not refer to them to predict patients' conditions.

Nevertheless, they referred to criteria such as the Braden Scale to determine the risk of pressure ulcers and the Morse Fall Risk Assessment to predict certain conditions. Kim et al. (39) point out in their study that although the warning scores of these criteria may be useful in identifying at-risk patients, direct examinations and ongoing surveillance by health care providers should be emphasized (39).

Nurses who participated in the present study also stated that a nurse should always be “attentive” in order to be ahead of the patient in terms of nursing care by projecting the patient's condition to prepare the environment for emergencies to control the situation. Through environmental foresight and predicting the patient's status into the near future, a nurse anticipates sudden changes in circumstances contrary to the expected conditions and predicts work shift conditions.

### Limitation and strengths of the study

Participants may, in some cases, have stated what they knew rather than what they did; attempts were made to interview informed people and those who were willing to participate in the study, and the observation method was also used to collect data. On the other hand, it is difficult to comment on our findings due to the lack of similar studies for comparison.

Despite these limitations, we argue that the insights and knowledge gained from this study can be useful for future practice, education, and research. For future studies, to develop and explain this concept, it is suggested that similar studies be conducted on other members of the health care team and other health settings.

## Conclusion

Findings obtained according to Endsley's SA model further developed this cognitive concept among ICU nurses. This study provides a practical and contextual definition of SA in ICU nurses. SA was experienced in ICU nurses as follows; first, a nurse understands the elements in the clinical environment, including human, physical and organizational aspects of the environment, through perception of clinical cues of the patient and their environment. Then, using the sense of salience, i.e., giving importance to the cues and analyzing and interpreting the received cues, the nurse becomes able to comprehend the patient's current situation. Finally, through environmental foresight and the prediction of patients' status in the near future, they can project the future situation.

## Data availability statement

The original contributions presented in the study are included in the article/Supplementary material, further inquiries can be directed to the corresponding author.

## Ethics statement

The studies involving human participants were reviewed and approved by Shahid Beheshti University of Medical Sciences. The patients/participants provided their written informed consent to participate in this study.

## Author contributions

RE and AE supervised this study. RE, AE, and CG designed the study and cooperated in composing, reviewing, and correcting the written version. AE and CG prepared the interview guide. CG conducted the interviews. RE and CG analyzed the interviews. All authors contributed to the article and approved the submitted version.

## Conflict of interest

The authors declare that the research was conducted in the absence of any commercial or financial relationships that could be construed as a potential conflict of interest.

## Publisher's note

All claims expressed in this article are solely those of the authors and do not necessarily represent those of their affiliated organizations, or those of the publisher, the editors and the reviewers. Any product that may be evaluated in this article, or claim that may be made by its manufacturer, is not guaranteed or endorsed by the publisher.
